# Short-term and mid-term evaluation of three types of minimally invasive lumbar fusion surgery for treatment of L4/L5 degenerative spondylolisthesis

**DOI:** 10.1038/s41598-024-54970-5

**Published:** 2024-02-21

**Authors:** Zhaojun Song, Zhi Zhang, Jiazhuang Zheng, Kai Zhang, Fandong Wang, Maobo Ran, Juan Luo

**Affiliations:** 1 Spine Surgery Department of Suining Central Hospital, Sichuan, People’s Republic of China; 2 Operation Room of Suining Central Hospital, Sichuan, People’s Republic of China; 3 Medical Record Department of Suining Central Hospital, Sichuan, People’s Republic of China

**Keywords:** Lumbar degenerative spondylolisthesis, MIS-TLIF, OLIF, Endo-TLIF, Neuromuscular disease, Neuromuscular disease, Neurosurgery

## Abstract

This was a single-centre retrospective study. Minimally invasive techniques for transforaminal lumbar interbody fusion (MIS-TLIF), oblique lumbar interbody fusion (OLIF), and percutaneous endoscopic transforaminal lumbar interbody fusion (Endo-TLIF) have been extensively used for lumbar degenerative diseases. The present study analyses the short-term and mid-term clinical effects of the above three minimally invasive techniques on L4/L5 degenerative spondylolisthesis. In this retrospective study, 98 patients with L4/L5 degenerative spondylolisthesis received MIS-TLIF, 107 received OLIF, and 114 received Endo-TLIF. All patients were followed up for at least one year. We compared patient data, including age, sex, body mass index (BMI), Oswestry disability index (ODI), visual analogue scale of low back pain (VAS-B), visual analogue scale of leg pain (VAS-L), surgical time, blood loss, drainage volume, hospital stay, complications, and neurological status. Moreover, we performed imaging evaluations, including lumbar lordosis angle (LLA), disc height (DH) and intervertebral fusion status. No significant differences were noted in age, sex, BMI, preoperative ODI, preoperative VAS-B, preoperative VAS-L, preoperative LLA, or preoperative DH. Patients who underwent OLIF had significantly decreased blood loss, a lower drainage volume, and a shorter hospital stay than those who underwent MIS-TLIF or Endo-TLIF (*P* < 0.05). The VAS-B in the OLIF group significantly decreased compared with in the MIS-TLIF and Endo-TLIF groups at 6 and 12 months postoperatively (*P* < 0.05). The VAS-L in the Endo-TLIF group significantly decreased compared with that in the MIS-TLIF and OLIF groups at 6 months postoperatively (*P* < 0.05). The ODI in the OLIF group was significantly better than that in the MIS-TLIF and Endo-TLIF groups at 6 months postoperatively (*P* < 0.05). No statistically significant differences in the incidence of complications and healthcare cost were found among the three groups. Follow-up LLA and DH changes were significantly lower in the OLIF group than in the other groups (*P* < 0.05). The intervertebral fusion rate was significantly higher in the OLIF group than in the other groups at 6 and 12 months postoperatively (*P* < 0.05). In conclusion, while MIS-TLIF, OLIF, and Endo-TLIF techniques can effectively treat patients with L4/5 degenerative spondylolisthesis, OLIF has more benefits, including less operative blood loss, a shorter hospital stay, a smaller drainage volume, efficacy for back pain, effective maintenance of lumbar lordosis angle and disc height, and a higher fusion rate. OLIF should be the preferred surgical treatment for patients with L4/5 degenerative spondylolisthesis.

## Introduction

Lumbar spondylolisthesis is a prevalent chronic disorder of the lumbar spine with a global incidence of approximately 6%^1^. The cause of lumbar spondylolisthesis can be degenerative, isthmic defect, dysplasia, traumatic, or pathological; among these, degenerative lumbar spondylolisthesis is the most prevalent. Lumbar spondylolisthesis with sagittal imbalance is often treated through lumbar fusion surgery^2^, which was first used more than 100 years ago. Conventional lumbar fusion surgery involves anterior lumbar interbody fusion (ALIF), posterior lumbar interbody fusion (PLIF), and transforaminal lumbar interbody fusion (TLIF), among other advanced technologies. With the advent of technology and advanced equipment, minimally invasive spinal surgical techniques have been widely applied. The minimally invasive transforaminal lumbar interbody fusion (MIS-TLIF) technique was introduced in 2002^3^^,^^4^ and is an effective surgical procedure for lumbar spondylolisthesis^5–7^. Oblique lumbar interbody fusion (OLIF) was first reported in 2012 by Silvestre^8^. It yields indirect neural decompression and preserves the lamina, paravertebral muscles, and facet joints through the anatomical space between the abdominal aorta and significant muscles of the psoas. Unlike conventional posterior surgery, OLIF has the benefits of less damage and bleeding, a lower rate of nerve injury, and a faster recovery rate^9–12^. In recent years, percutaneous endoscopic transforaminal lumbar interbody fusion (Endo-TLIF) has been successfully attempted to treat patients with lumbar spondylolisthesis^13–15^. The endo-TLIF technique originated from the percutaneous endoscopic lumbar discectomy technique and integrated endoscopic visualization, a conventional or expandable cage, and the interbody fusion technique. Several researchers have reported the predominant benefits of Endo-TLIF, including small incisions, less stripping of the paraspinal muscles, minimal blood loss, and rapid postoperative recovery^16–18^.

Although MIS-TLIF, OLIF, and Endo-TLIF technologies have been extensively applied for lumbar degenerative diseases, there are few reports on systematic analysis, evaluation, and application in treating degenerative lumbar spondylolisthesis. Therefore, this work aimed to evaluate and compare the short-term and mid-term postoperative clinical effects of the above three minimally invasive L4/L5 degenerative spondylolisthesis techniques. Moreover, we sought to provide spine surgeons and patients with precise early outcomes of MIS-TLIF, OLIF, and Endo-TLIF and a relevant theoretical basis for a suitable operation method.

## Methods

### Patients

This study was approved by the Ethics Committee of the Suining Central Hospital and informed consent was obtained from all subjects. All procedures performed in studies involving human participants were in accordance with the ethical standards of the institutional and national research committee and with the 1964 Helsinki declaration and its later amendments or comparable ethical standards. The study included 319 patients with L4/L5 degenerative spondylolisthesis who received minimally invasive lumbar fusion surgery. In total, 98 patients received MIS-TLIF (WEGO Inc. Shandong, China, defined as the MIS-TLIF group), 107 OLIF (WEGO Inc. Shandong, China, defined as the OLIF group), and 114 patients Endo-TLIF (WEGO Inc. Shandong, China and SPINENDOS, Munich, Germany, defined as the Endo-TLIF group) between March 2018 and April 2022.

The inclusion criteria included the following: (1) patients with single-level (L4/L5) Meyerding grade I and II degenerative spondylolisthesis; (2) diagnosis by lumbar flexion–extension stress lateral radiographs, computed tomography (CT), and magnetic resonance imaging (MRI) of the lumbar vertebra disc; (3) neurological examination, including intermittent neurogenic claudication, clinical sciatica, or mechanical low back pain with segmental instability; and (4) OLIF as the preferred surgical treatment for patients with low back pain or dynamic radicular pain such as neurogenic intermittent claudication pain as the cardinal symptom. MIS-TLIF or Endo-TLIF is the preferred surgical treatment for patients with static radicular pain as the initial symptom^19^. (5) All patients underwent an operation for the first time; (6) invalidation of conservative treatment for > 3 months, as well as operation indicators and the treatment expected to be effective; (7) clinical data were complete, and patients were followed up for at least one year. On the other hand, the exclusion criteria included (1) patients who underwent revision surgery; (2) patients who had undergone previous lumbar fusion surgery; (3) patients who needed lumbar fusion for more than one level or other levels than L4/L5; and (4) isthmic defect, dysplasia, trauma, tumour, or nondegenerative pathologies at L4/L5 requiring fusion.

We performed preoperative examinations, including routine blood tests, blood biochemistry analysis, blood electrolytes, coagulation evaluation, pretransfusion tests, blood type, chest X-ray, electrocardiogram, lumbar anterior–posterior (A-P) and lateral radiographs, lumbar flexion–extension stress lateral radiographs, CT and MRI of the lumbar spine.

**Figure 1:**
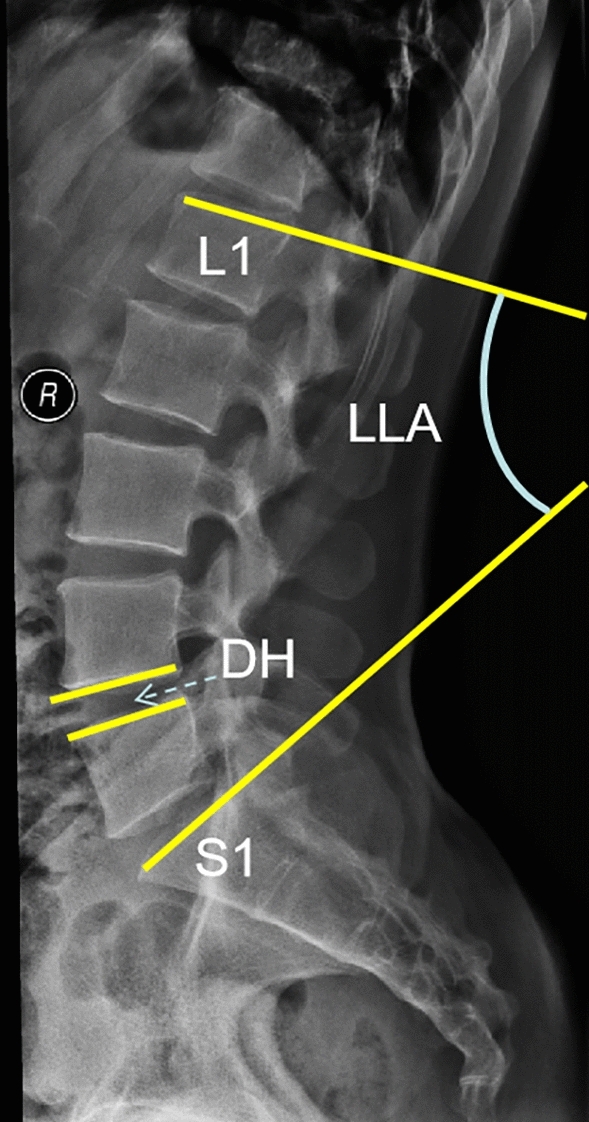
Method for measuring the LLA and DH.

The baseline characteristics of the three groups, including age, sex, body mass index (BMI), preoperative Oswestry disability index (ODI), preoperative visual analogue scale of low back pain (VAS-B), preoperative visual analogue scale of leg pain (VAS-L), preoperative lumbar lordosis angle (LLA), and preoperative disc height (DH), were not different (Table 1). DH was defined as the distance of the upper endplate to the lower endplate of two vertebral bodies on the lumbar lateral film. LLA was defined as the included angle of the upper endplate of the 1st lumbar vertebra and the upper endplate of the 1st sacral vertebra (Fig. 1).

**Table 1:** Comparison of the baseline information of the three groups.

Groups	MIS-TLIF	OLIF	Endo-TLIF	*P*-value
Male/female	45/ 53	51/ 56	60/ 54	0.158
Age (years)	64.1 ± 8.5	60.9 ± 9.3	63.3 ± 8.8	0.251
BMI	23.2 ± 3.3	23.6 ± 2.8	23.8 ± 2.1	0.956
Pre VAS-B	5.4 ± 1.2	4.9 ± 1.5	5.5 ± 0.9	0.182
Pre VAS-L	5.6 ± 1.1	5.4 ± 1.5	5.9 ± 1.7	0.176
Pre ODI (%)	55.7 ± 8.6	57.1 ± 9.7	58.5 ± 8.9	0.638
Pre LLA(°)	25.8 ± 5.3	24.8 ± 4.8	24.2 ± 4.6	0.632
Pre DH(mm)	9.1 ± 2.2	9.4 ± 1.9	9.5 ± 2.5	0.289

MIS-TLIF: minimally invasive technique for transforaminal lumbar interbody fusion; OLIF: oblique lumbarinter body fusion; Endo-TLIF: percutaneous endoscopic transforaminal lumbar interbody fusion; BMI: body mass index: Pre: preoperative; VAS-B: visual analog scale of low back pain; VAS-L: visual analog scale of leg pain; ODI: Oswestry disability index; LLA: lumbar lordosis angle; DH: disc height.

### Surgical technique

**Figure 2:**
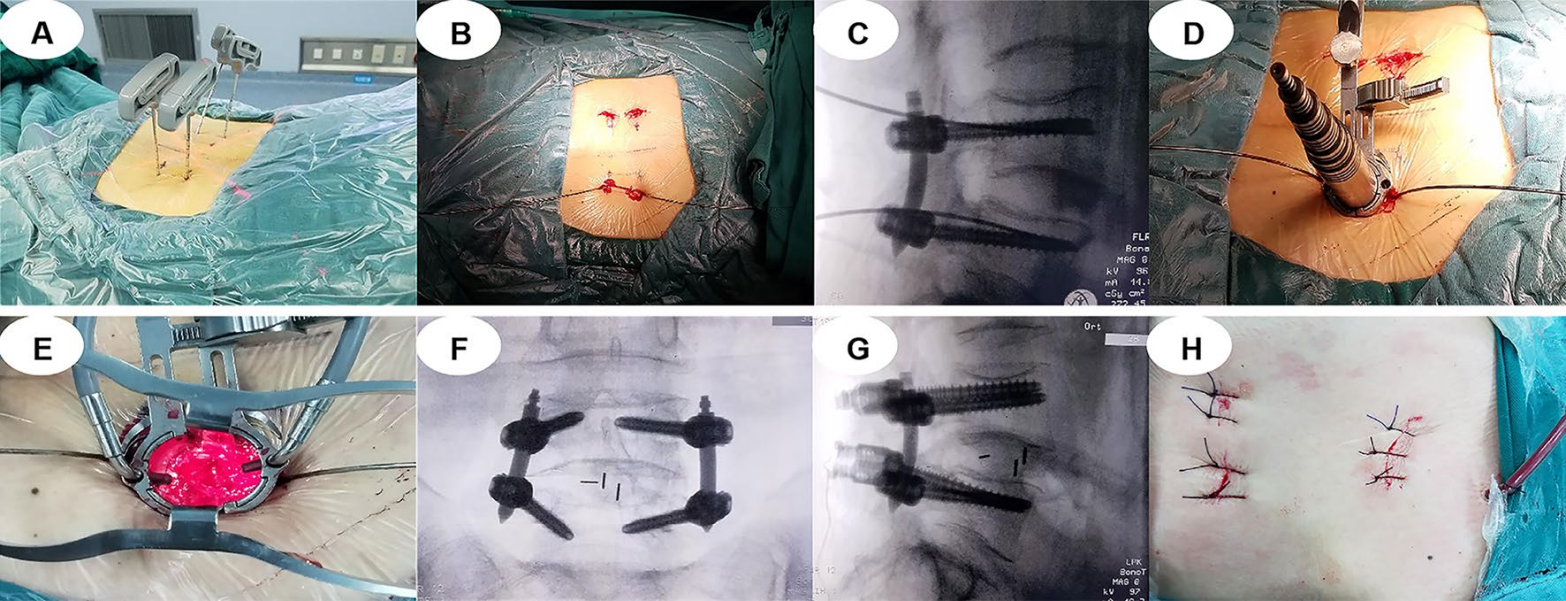
A typical MIS-TLIF case. (**A**–**C**) Posterior unilateral percutaneous pedicle screw fixation and vertebral reduction via fluoroscopy. (**D**) Insertion of the expansion tube and installation of the quadrant working channel. (**E**) Decompression and fusion under the working channel. (**F**–**G**) Position of the cage and percutaneous pedicle screws in anteroposterior and lateral X-ray views. (**H**) Incision and drainage tube.

MIS-TLIF group (Fig. 2): Patients under general anaesthesia were placed in the prone position with ECG and neurophysiologic monitoring. The C-arm marked the L4/L5 segment and pedicles, two posterior unilateral pedicle screws (WEGO Inc. Shandong, China) were inserted via fluoroscopy, and guide wires were reserved on the symptomatic side. A 3.5-cm incision was made at 6 cm from the posterior median line; an expansion tube was systematically inserted and installed in the quadrant working channel. The facet joint was exposed, and part of the superior and inferior articular process and ligamentum flavum was removed, exposing and protecting the dural sac and nerve root. The herniated disc and cartilaginous endplate were removed until bleeding cancellous bone was exposed. After artificial bone grafting (Shanghai Rebone Biomaterials Co., Ltd, China), a right-sized PEEK intervertebral cage (WEGO Inc. Shandong, China) was inserted via the working channel; subsequently, the nerve root was loosened and reinspected. Two ipsilateral pedicle screws were inserted, and then the two angled titanium rods were placed over the screws before the screw heads were tightened. The surgical site was washed with normal saline; then, a drainage tube was placed in the wound, and the skin was sutured.

**Figure 3:**
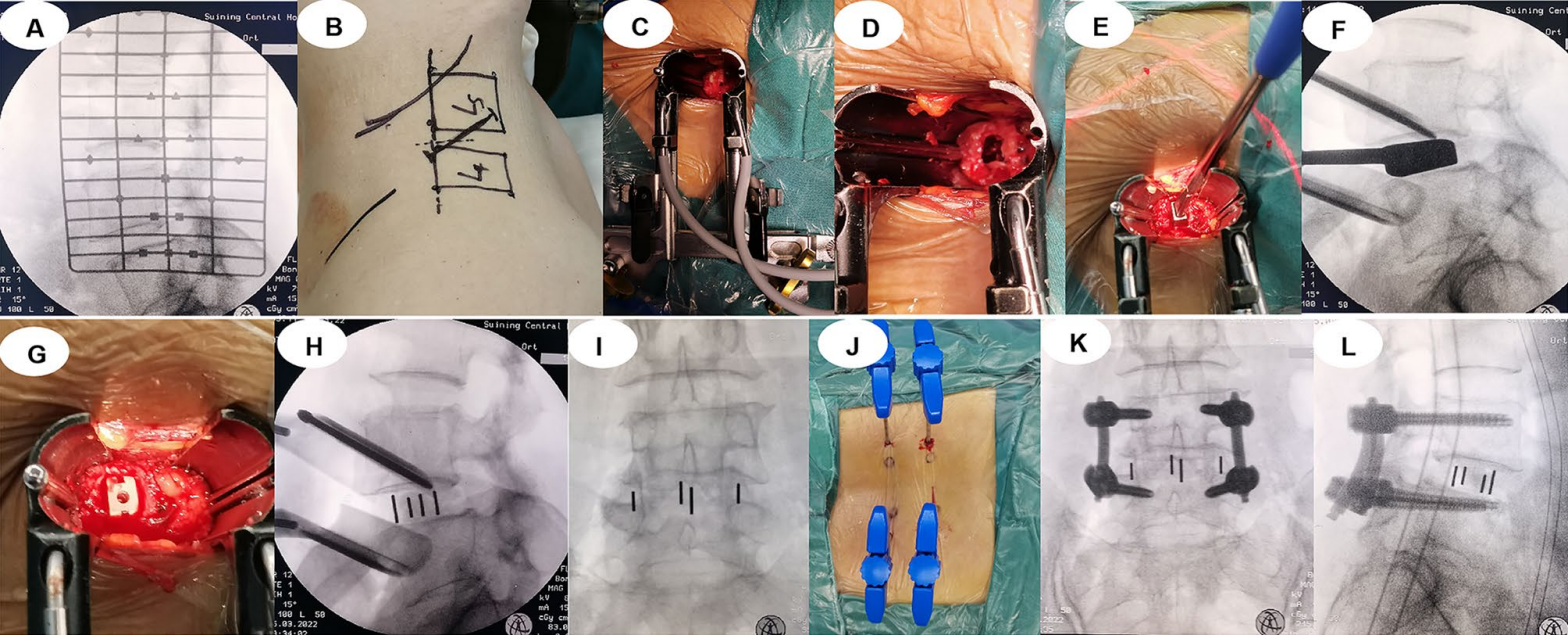
A typical OLIF case. (**A**, **B**) Marks of the L4/L5 segment and incision. (**C**) Exposure of the operative field and installation of the working passage. (**D**) Discectomy and endplate preparation. (**E**, **F**) Assessing the interbody cage. (**G**–**I**) Implantation of the cage. (**J**–**L**) Position of the cage and percutaneous pedicle screws in anteroposterior and lateral X-ray views.

OLIF group (Fig. 3): Patients under general anaesthesia were placed in the right lateral position with ECG monitoring. The C-arm marked the L4/L5 segment. A 4-cm left lateral incision was made above the iliac crest. Afterwards, the abdominal muscles, including the external oblique, internal oblique, and transverse abdominal muscles, were bluntly split, and the retroperitoneum space was entered. The retractor was used to anteriorly sweep the peritoneum, whereas the psoas major muscle was posteriorly retracted. A working passage was placed between the abdominal aorta and psoas major muscles with direct visualization of the disc and centrum. Discectomy and endplate preparation were performed through the passage, and then an appropriate PEEK cage (WEGO Inc. Shandong, China) with a 5° lordosis angle filled with artificial bone (Shanghai Rebone Biomaterials Co., Ltd, China) was inserted into the disc space after assessing interbody cages. The surgical site was washed with normal saline, a rubber drainage tube was placed at the wound, and the skin was sutured. The patient was placed in a prone position for minimally invasive percutaneous pedicle screw fixation (WEGO Inc. Shandong, China).

**Figure 4:**
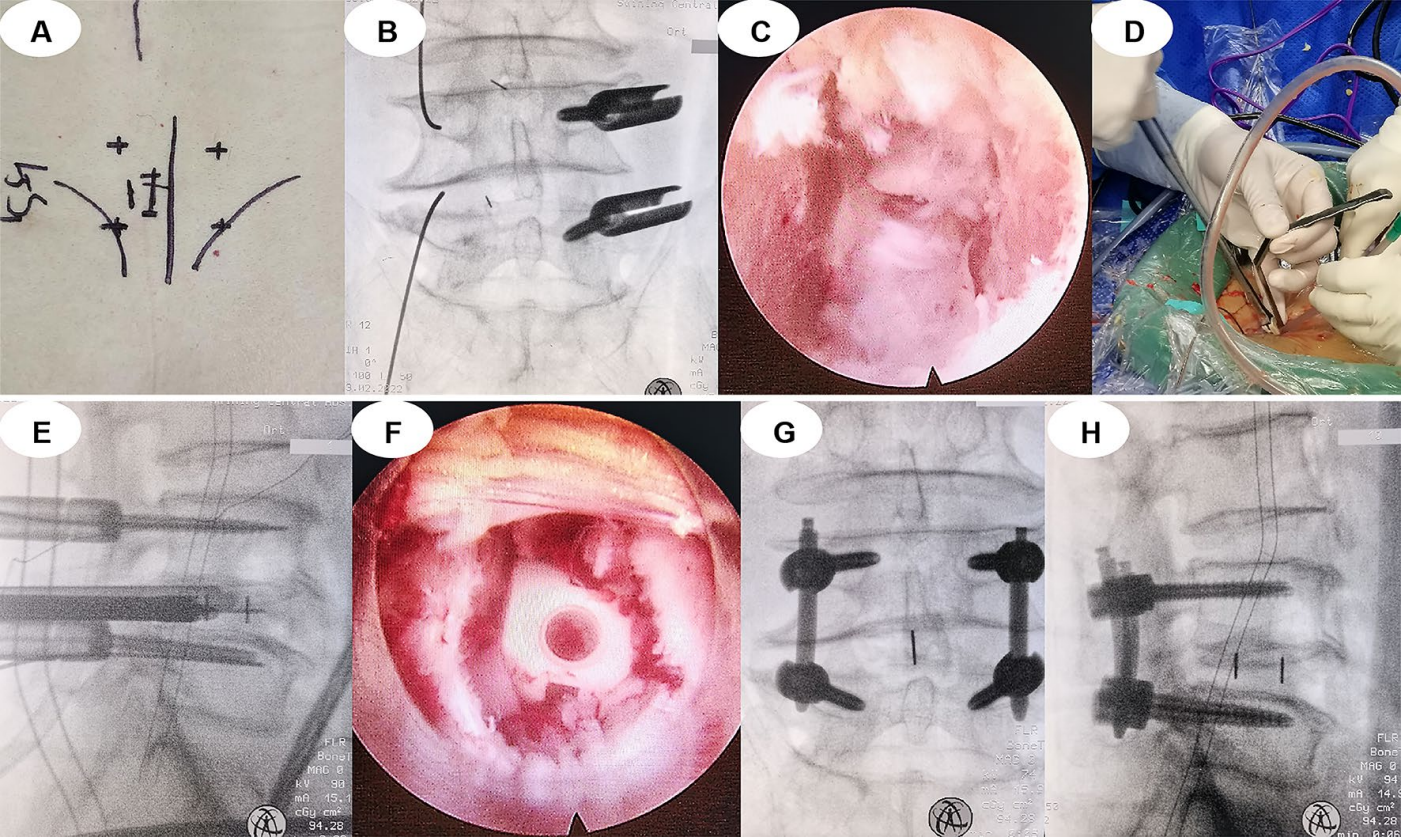
A typical Endo-TLIF case. (**A**) Marks of the L4/L5 vertebral pedicle and incision. (**B**) Posterior unilateral percutaneous pedicle screw fixation. (**C**) Discectomy, endplate preparation and nerve root decompression under endoscopy. (**D**–**F**) Implantation of the cage. (**H**) Position of cage and pedicle screws in anteroposterior and lateral X-ray views.

Endo-TLIF group (Fig. 4): Patients under general anaesthesia were placed in a prone position with ECG and neurophysiologic monitoring. The C-arm marked the L4/L5 segment and pedicles, a posterior unilateral pedicle screw (WEGO Inc. Shandong, China) was inserted via fluoroscopy, and guide wires were reserved on the symptomatic side. A 1.5-cm incision was made 2 cm from the posterior median line for endoscopic working channel insertion (SPINENDOS, Munich, Germany). Part of the superior and inferior articular processes was removed for foraminoplasty and laminectomy with a special full-see osteotome. Discectomy, endplate preparation, nerve root decompression, and bone graft were performed under endoscopy. A special nerve root retractor was inserted to protect the dural sac and nerve root outside the endoscopic working channel before removing the endoscopic working channel. An appropriate PEEK cage (WEGO Inc. Shandong, China) filled with artificial bone (Shanghai Rebone Biomaterials Co., Ltd, China) was inserted via the retractor for lumbar interbody fusion, and the location of the cage was monitored via fluoroscopy. The nerve root retractor was used to reinsert the endoscopic working channel, and the nerve root was once more investigated under endoscopy. A rubber drainage tube was placed at the wound, and the skin was sutured.

### Clinical evaluation

All patients were followed up for at least one year. We summarized, calculated, and compared their information, including surgical time, blood loss, drainage volume, drainage time, hospital stay, and complications. All patients were clinically assessed using VAS-B and VAS-L, ranging from no pain (0 points) to the worst pain imaginable (10 points). The patients were functionally assessed based on the ODI. VAS-B and VAS-L evaluations were performed preoperatively and at 6 months and 12 months postoperatively. ODI assessments were conducted preoperatively and at the 6-month and 12-month follow-ups.

Standing frontal and lateral radiographs of the lumbar spine were postoperatively obtained at 1 week and at 3, 6, and 12 months. Measurements were recorded for LLA and DH. A single investigator measured LLA and DH and recorded their changes between preoperation, postoperation, and follow-up. Postoperative fusion status was assessed by CT at 6 to 12 months after surgery. Continuous bone trabecular bridging between intervertebral bodies is considered the gold standard of spinal intervertebral fusion^20^.

### Statistical analysis

Statistical Product and Service Solutions (SPSS) 19.0 statistical software (IBM, Armonk, NY, USA) was used for statistical analyses, and measurement data were recorded as the mean ± standard deviation (SD). Single-factor variance analysis was used to compare differences in measurement data among the three groups. The chi-square test was used to analyse enumeration data. A *P* value < 0.05 was considered statistically significant.

## Results

### Comparison of surgical results

A total of 98 patients were followed up after MIS-TLIF, 107 patients after OLIF, and 114 patients after Endo-TLIF for at least 1 year. As shown in Table 2, no statistically significant differences were noted among the three groups with regard to operating time (MIS-TLIF group: 116.5 ± 21.2 min, OLIF group: 103.4 ± 28.5 min, Endo-TLIF group: 107.4 ± 22.9 min, *P* = 0.558). Patients in the OLIF group had less blood loss (MIS-TLIF group: 104.6 ± 38.1 ml, OLIF group: 75.1 ± 31.4 ml, Endo-TLIF group: 96.5 ± 36.2 ml, *P* = 0.036), a shorter hospital stay (MIS-TLIF group: 11.6 ± 4.2 days, OLIF group: 7.5 ± 3.6 days, Endo-TLIF group: 10.2 ± 4.3 days, *P* = 0.023) and a lower volume of drainage (MIS-TLIF group: 152.5 ± 53.4 ml, OLIF group: 22.4 ± 12.6 ml, Endo-TLIF group: 114.2 ± 37.1 ml, *P* = 0.031) than those in the other two groups. And no statistically significant differences were found among the three groups in healthcare cost (MIS-TLIF group: 70,908.5 ± 5577.8 CNY, OLIF group: 75,592.0 ± 4850.3 CNY, Endo-TLIF group: 78,367.4 ± 13,022.3 CNY, *P* = 0.169).

**Table 2:** Comparison of surgical results and healthcare cost between the three groups.

Groups	MIS-TLIF	OLIF	Endo-TLIF	*P*-value
Operating time (min)	116.5 ± 21.2	103.4 ± 28.5	107.4 ± 22.9	0.558
Blood loss (ml)	104.6 ± 38.1	75.1 ± 31.4	96.5 ± 36.2	0.036
Hospital stay (days)	11.6 ± 4.2	7.5 ± 3.6	10.2 ± 4.3	0.023
Volume of drainage (ml)	152.5 ± 53.4	22.4 ± 12.6	114.2 ± 37.1	0.031
Complications (n)	9/98	10/107	8/114	0.786
Healthcare cost	70,908.5 ± 5577.8	75,592.0 ± 4850.3	78,367.4 ± 13,022.3	0.169

Healthcare cost was compared in China Yuan (CNY).

### Functional outcomes and ODI scores

The VSA score and ODI evaluated postoperative functional outcomes. As shown in Table 3, VAS-B scores were 1.4 ± 0.9 for the MIS-TLIF group, 1.0 ± 0.7 for the OLIF group, and 1.5 ± 1.3 for the Endo-TLIF group (*P* = 0.035) at 6 months. Among the three groups, the VAS-L relief level was significantly better in the Endo-TLIF group at 6 months postoperatively (MIS-TLIF group: 1.3 ± 0.9, OLIF group: 1.5 ± 1.1, Endo-TLIF group: 1.1 ± 0.8, *P* = 0.017). VAS-B scores were 1.1 ± 0.7 for the MIS-TLIF group, 0.6 ± 0.4 for the OLIF group, and 0.9 ± 0.6 for the Endo-TLIF group (*P* = 0.044) and VAS-L scores 0.8 ± 0.9 for the MIS-TLIF group, 0.9 ± 0.5 for the OLIF group, and 0.6 ± 0.8 for the Endo-TLIF group (*P* = 0.105) at 12 months postoperatively.

**Table 3:** Comparison of VAS scores between the three groups.

Groups	Pre VAS-B	Pre VAS-L	VAS-B after 6 months	VAS-L after 6 months	VAS-B after 12 months	VAS-L after 12 months
MIS-TLIF	5.4 ± 1.2	5.6 ± 1.1	1.4 ± 0.9	1.3 ± 0.9	1.1 ± 0.7	0.8 ± 0.9
OLIF	4.9 ± 1.5	5.4 ± 1.5	1.0 ± 0.7	1.5 ± 1.1	0.6 ± 0.4	0.9 ± 0.5
Endo-TLIF	5.5 ± 0.9	5.9 ± 1.7	1.5 ± 1.3	1.1 ± 0.8	0.9 ± 0.6	0.6 ± 0.8
P-value	0.182	0.176	0.035	0.017	0.044	0.105

As shown in Table 4, ODI improvement was significantly better in the OLIF group at 6 months postoperatively (MIS-TLIF group: 38.2 ± 5.1, OLIF group: 31.4 ± 6.6, Endo-TLIF group: 32.9 ± 5.7, *P* = 0.039). The ODI was 16.1 ± 4.7 for the MIS-TLIF group, 14.4 ± 5.1 for the OLIF group, and 15.7 ± 5.9 for the Endo-TLIF group (*P* = 0.095) at 12 months postoperatively.

**Table 4:** Comparison of ODI (%) scores between the three groups.

Groups	Pre ODI	ODI after 6 months	ODI after 12 months
MIS-TLIF	55.7 ± 8.6	38.2 ± 5.1	16.1 ± 4.7
OLIF	57.1 ± 9.7	31.4 ± 6.6	14.4 ± 5.1
Endo-TLIF	58.5 ± 8.9	32.9 ± 5.7	15.7 ± 5.9
*P*-value	0.638	0.039	0.095

### Comparison of treatment effects and complications

**Figure 5:**
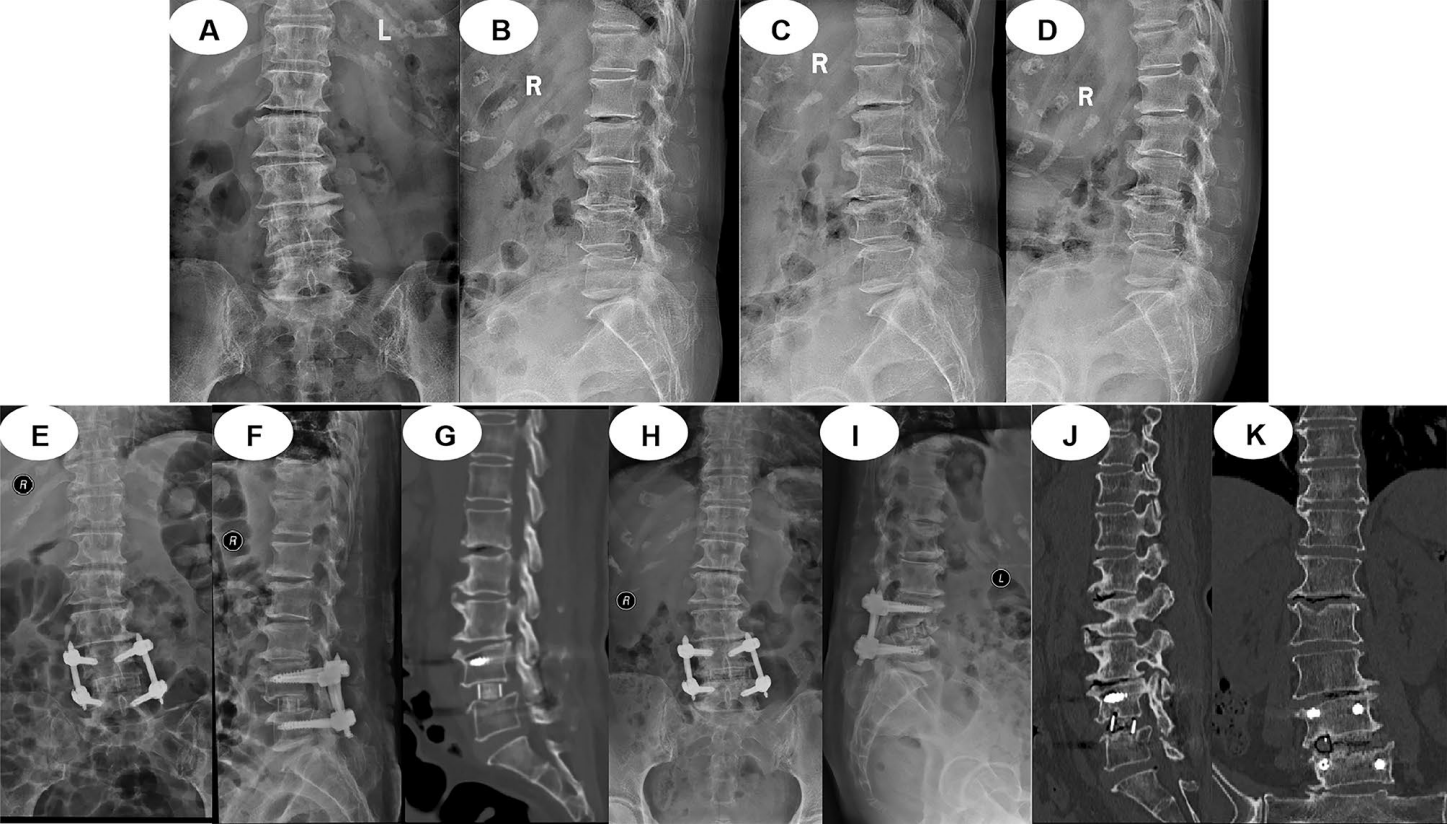
A 74-year-old woman had cage subsidence after OLIF. Preoperative lumbar spine anteroposterior and lateral radiographs (**A**, **B**), lumbar flexion–extension stress lateral radiographs (**C**, **D**). X-ray radiographs (**E**, **F**) and CT (**G**) at 1 month postoperatively show that the cage was in a good position. X-ray radiographs (**H**, **I**) and CT (**J**, **K**) at 3 months postoperatively showed cage subsidence.

**Figure 6:**
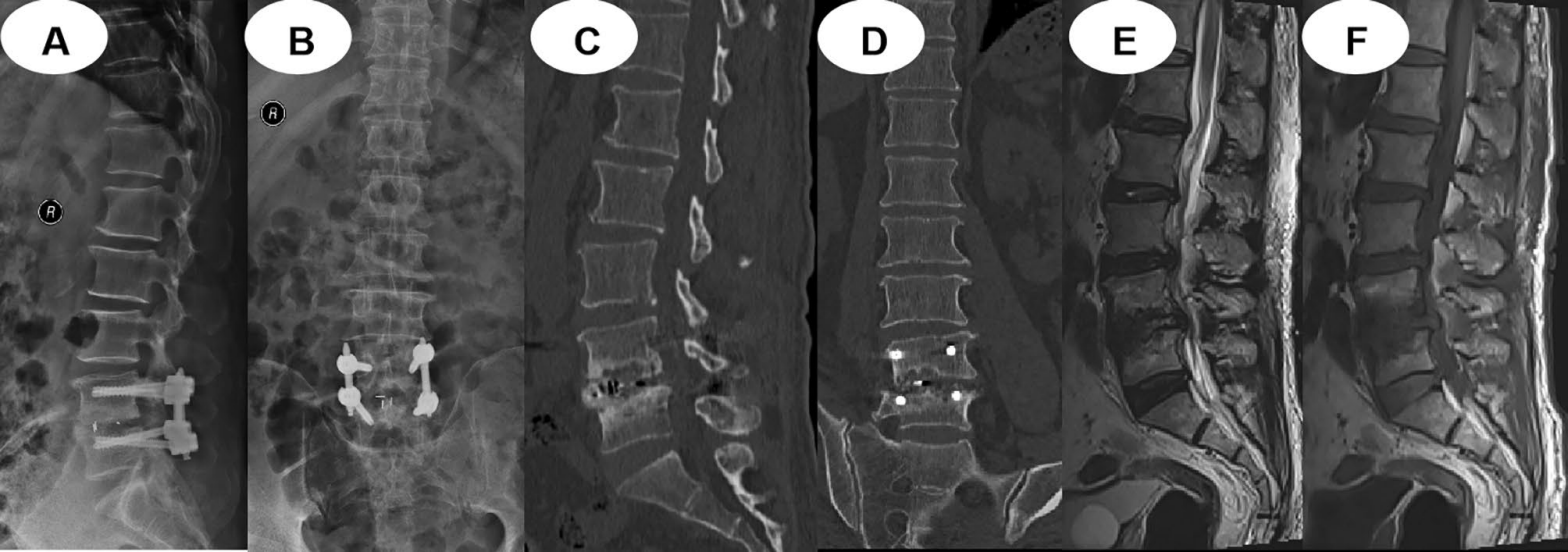
A 65-year-old man had intervertebral infection after Endo-TLIF. A-P and lateral plain film of the lumbar vertebra showed narrowing of the intervertebral space (**A**, **B**); sagittal and coronal CT showed that the vertebrae were rough, blurry, broken and sclerotic (**C**, **D**); sagittal T1- and T2-weighted MRI showed intervertebral infection (**E**, **F**) at 6 months after surgery.

Nine patients in the MIS-TLIF group, 10 in the OLIF group, and 8 in the Endo-TLIF group experienced complications (Table 2). No statistically significant differences were found among the three groups for the incidence of complications (*P* = 0.786). Complications in the MIS-TLIF group included 2 cases of pulmonary infection, 2 cases of urinary tract infection, 1 case of abdominal distension, 2 cases of cerebrospinal fluid leakage, and 2 cases of segmental nerve root palsy. Complications in the OLIF group included 2 cases of cage subsidence (Fig. 5), 1 case of nerve root palsy, 3 cases of abdominal distension, and 4 cases of endplate fracture; complications in the Endo-TLIF group included 4 cases of cerebrospinal fluid leakage, 2 cases of segmental nerve root palsy, 1 case of abdominal distension, and 1 case of intervertebral infection (Fig. 6).

### Postoperative imaging evaluation

Imaging evaluation included preoperative and postoperative changes in lumbar LLA and DH. As shown in Table 5, LLA increased from a mean of 25.8° preoperatively to 34.3° postoperatively in the MIS-TLIF group. The OLIF group showed an increase from a mean of 24.8° preoperatively to 35.2° postoperatively, and the Endo-TLIF group showed an increase from a mean of 24.2° preoperatively to 33.9° postoperatively. The LLA increase was not significantly different (*P* = 0.359). Notably, LLA in the OLIF group was higher than that in the other two groups at 12 months after surgery (MIS-TLIF group: 32.5° ± 4.3; OLIF group: 34.4° ± 4.1; Endo-TLIF group: 32.2° ± 3.9; *P* = 0.037). The follow-up LLA decreased in all the groups compared to that at 3 days after surgery, and the reduction in LLA in the OLIF group was smallest (MIS-TLIF group: 1.8°; OLIF group: 0.8°; Endo-TLIF group: 1.7°; *P* = 0.012). As shown in Table 6, the DH change (mm) at 3 days after surgery was 2.4 ± 1.1 in the MIS-TLIF group, 3.2 ± 1.6 in the OLIF group, and 2.3 ± 1.8 in the Endo-TLIF group (*P* = 0.041). Follow-up DH changes were lower in the OLIF group than in the other groups (MIS-TLIF group: 1.3 mm; OLIF group: 1.0 mm; Endo-TLIF group: 1.2 mm; *P* = 0.018).

**Table 5:** LLA(°)changes between the three groups.

Groups	Pre LLA	Post LLA	F/U LLA	Post LLA—Pre LLA	F/U LLA—Post LLA
MIS-TLIF	25.8 ± 5.3	34.3 ± 5.6	32.5 ± 4.3	9.2 ± 2.3	–1.8 ± 1.1
OLIF	24.8 ± 4.8	35.2 ± 5.2	34.4 ± 4.1	10.4 ± 2.1	–0.8 ± 1.7
Endo-TLIF	24.2 ± 4.6	33.9 ± 4.4	32.2 ± 3.9	9.7 ± 2.7	–1.7 ± 1.5
P-value	0.632	0.359	0.037	0.053	0.012

LLA: lumbar lordosis angle; Pre: Preoperative; Post: post-operative (3 days after surgery); F/U: follow-up (12 months after surgery).

**Table 6:** DH(mm)changes between the three groups.

Groups	Pre DH	Post DH	F/U DH	Post DH—Pre DH	F/U DH—Post DH
MIS-TLIF	9.1 ± 2.2	11.5 ± 1.6	10.2 ± 1.7	2.4 ± 1.1	–1.3 ± 0.4
OLIF	9.4 ± 1.9	12.6 ± 1.1	11.5 ± 1.6	3.2 ± 1.6	–1.0 ± 0.6
Endo-TLIF	9.5 ± 2.5	11.8 ± 1.5	10.6 ± 1.1	2.3 ± 1.8	–1.2 ± 0.4
P-value	0.289	0.195	0.031	0.041	0.018

DH: disc height; Pre: Preoperative; Post:post-operative (3 days after surgery); F/U: follow-up (12 months after surgery).

**Figure 7:**
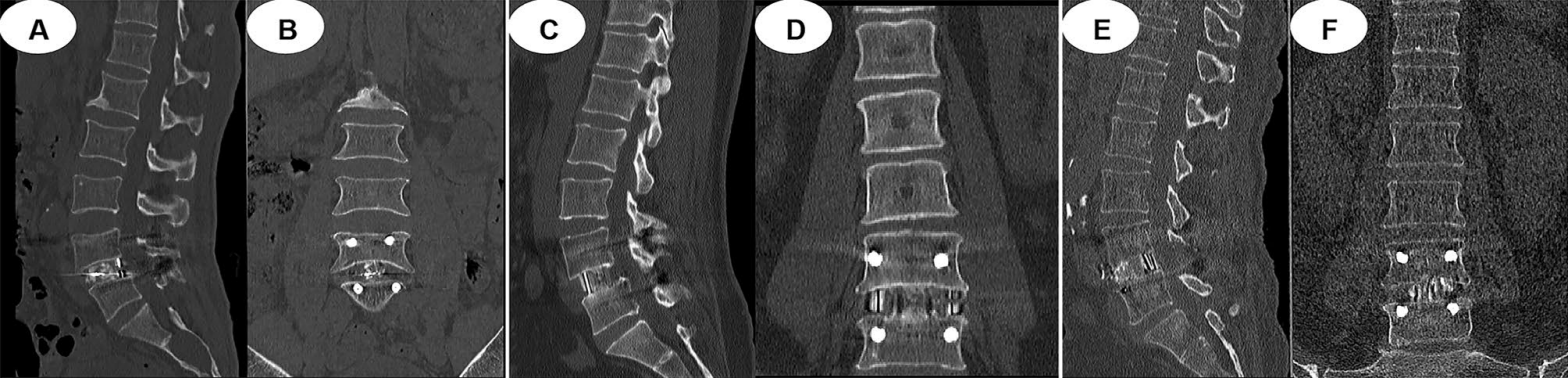
(**A**, **B**) CT at 12 months postoperatively showing intervertebral fusion after MIS-TLIF. (**C**, **D**) CT at 12 months postoperatively showing intervertebral fusion after OLIF. (**E**, **F**) CT at 12 months postoperatively showing intervertebral fusion after Endo-TLIF.

Regarding fusion status, 59 patients (60.2%) in the MIS-TLIF group, 81 patients (75.7%) in the OLIF group, and 68 patients (59.6%) in the Endo-TLIF group showed complete union at 6 months after the operation (*P* = 0.020). Eighty-four of 98 patients (85.7%) in the MIS-TLIF group, 101 of 107 patients (94.4%) in the OLIF group, and 96 of 114 patients (84.2%) in the Endo-TLIF group showed complete union after the one-year follow-up (*P* = 0.045; Fig. 7).

## Discussion

Over the past 2 decades, minimally invasive spinal techniques have significantly improved treatment of degenerative spinal diseases. The most predominant areas include minimally invasive spinal reconstruction and minimally invasive decompression technologies represented by spinal endoscopy. Technological advancement, tool improvement, and patient demand are the most important factors promoting development of minimally invasive spinal surgery. Long-term outcomes, recommendations by the surgeon, and complication risk are critical criteria when selecting between open and minimally invasive spine surgery. Most patients (80%) prefer a minimally invasive technique if they need spine surgery^21^.

MIS-TLIF, OLIF, and Endo-TLIF are the three types of minimally invasive lumbar fusion surgery techniques extensively applied in lumbar degenerative diseases. However, studies that systematically analyse and evaluate application of these three technologies for treatment of degenerative lumbar spondylolisthesis are unavailable. Herein, we compare early clinical results of three types of minimally invasive lumbar fusion surgery in L4/L5 degenerative spondylolisthesis. All cases were followed up for at least 1 year.

For surgical findings, the blood loss, hospital stay, and drainage volume in the OLIF group were significantly less than those in the MIS-TLIF and Endo-TLIF groups, which is similar to previous literature^22–24^. In addition, the VAS-B relieving percentage in the OLIF group was significantly improved at 6 and 12 months postoperatively. This is related to the surgical path of OLIF. Unlike with the MIS-TLIF and Endo-TLIF techniques, in the OLIF approach, a working passage is placed between the major muscles of the abdominal aorta and psoas, and the lumbar posterior bone-ligament complex, including the paravertebral muscles, lamina, and facet joints, is not destroyed. Hiyama et al.^25^ also reported that the indirect decompression of lateral lumbar interbody fusion exhibited more significant improvement in low back pain, possibly because invasive surgery was not performed, which would have limited the stress on the posterior support elements such as the facet joints, lamina, and paraspinal muscles. This explains why OLIF has benefits of less bleeding, less drainage volume, shorter hospital stay and better VAS-B relieving percentage than the other two techniques. This also explains why the OLIF group had a better ODI in the early postoperative period than those who underwent the other two techniques. It should be noted that in all three minimally invasive procedures, a drainage tube was inserted, which has the advantages of reducing postoperative hematoma formation with all its complications and risk of wound infection^26,27^.

Although the VAS-L relief level was not significantly different at 12 months postoperatively, the VAS-L relief level was significantly higher in the Endo-TLIF group at 6 months postoperatively. Unlike in MIS-TLIF and OLIF, nerve root decompression under full-see endoscopy and continuous irrigation of normal saline may be the primary factors that alleviate nervous radical pain in Endo-TLIF. Similar to PELD, the nerve root can be protected by a working tube with a bevelled tip, which reduces the incidence of nerve root injury. A series of systematic review and meta-analyses indicated that Endo-TLIF showed advantages in less surgical trauma, faster recovery, and early postoperative relief of back pain compared to MIS-TLIF^28,29^. A prospective cohort study by Ao et al.^15^ also indicated that Endo-TLIF had advantages of less surgical trauma, less postoperative low-back pain, less hidden blood loss, and faster recovery, compared with MIS-TLIF. Similar results were obtained in our study even without statistical differences. Endo-TLIF group has better VAS-B relieving percentage than MIS-TLIF group at 6, 12 months postoperatively. In addition, Endo-TLIF reduced intraoperative blood loss and length of hospital stay compared to MIS-TLIF in our study. With smaller incision and less sof tissue traction, patients in Endo-TLIF group are likely to have better post-operative recovery and shorter hospital stay than patients in MIS-TLIF group. Moreover, Endo-TLIF allowed directly reaching the disc for achieving simultaneous decompression and fusion without excision of lamina, superior and inferior articular processes, and ligamentum flavum, like traditional MIS-TLIF technique. The massive posterior osseous structures was preserved with less muscle damage, reducing the bleeding of cancellous bone^15^. Together these factors might explain the reason that there were better VAS-B, less blood loss and shorter hospital stay in Endo-TLIF group compared to MIS-TLIF group.

Compared with previous studies^19,30,31^, longer mean time in hospitalization was observed in our study (MIS-TLIF group: 11.6 ± 4.2 days, OLIF group: 7.5 ± 3.6 days, Endo-TLIF group: 10.2 ± 4.3 days). The possible causes of longer hospital stay in our study are analyzed and listed as foilows: (1) Normally, patients were received rehabilitation trainnings for 7–10 days after operation. (2) Most of the patients requested discharge after removal sutures. (3) The mode of payment is another factor that affects the length of hospital stay for patients. The more patients with medical insurance there are, the longer the hospital stay will be. The medical insurance policy may weaken payment awareness of patients. Patients in the OLIF group had shorter hospital stays than those in the MIS-TLIF and Endo-TLIF groups. Unlike with the MIS-TLIF and Endo-TLIF techniques, in the OLIF approach, a working passage is placed between the major muscles of the abdominal aorta and psoas, and the lumbar posterior bone-ligament complex, including the paravertebral muscles, lamina, and facet joints, is not destroyed. Hiyama et al.^25^ reported that the indirect decompression of lateral lumbar interbody fusion had advantages of less surgical trauma, less postoperative low-back pain, and faster recovery. It's because that the lumbar posterior bone-ligament complex, including the paravertebral muscles, lamina, and facet joints, is not destroyed in the OLIF approach. Miscusi et al.^32^ also promoted that OLIF does not affect the function of the lumbar spine joints, allowing a faster return to physical activity.

In line with previous research results, the complication rate was 9.2% in the MIS-TLIF group, 9.3% in the OLIF group, and 7.0% in the Endo-TLIF group^8,11,13,33^. In our study, 4 cases of endplate fracture occurred, all of which were related to improper intervertebral space management. Two cases of cage subsidence were reported in the OLIF group (2.2%), according to the Malham GM method for classification of cage subsidence^34^, which was lower than that in the previous studies^35–37^. Reports indicate that the endplate preparation, implant material, and patient bone quality are related to cage sedimentation^36,38^. A finite element analyses have shown that preservation of the structural integrity of the vertebral endplate protects against sub-sidence, particularly in the cortical periphery, as does packing of the cage cavity with graft material^39^. A retrospective multivariable analysis indicated that the cage would sink when the excisional thickness of endplate achieve or approximate cut-off value of 3.7 mm^40^. Additionally, surgical technique has also been implicated, removing only the cartilaginous end plates and preserve bony end plate carefully to keep local strength and stiffness magnitudes could reduce the potential for cage subsidence^41,42^. Therefore, we believe that good endplate preparation and preservation of the bony endplate is a top priority for cage subsidence prevention. Patient factors increasing the risk of subsidence include high body mass index and low bone mineral density^43^. The two patients with cage subsidence appeared to have osteopenia. The preoperative T scores of lumbar bone mineral density with QCT for these two patients were -2.9 and -3.2. Therefore, we recommend that OLIF be avoided in those with osteoporosis. Endplate damage without posterior pedicle screw fixation accounts for sedimentation^44^. Liu et al.^10^ observed 18 cases with posterior fixation without cage sedimentation; the incidence of cage sedimentation was 3.85% in cases of unilateral fixation and 26.09% in stand-alone fixation cases during a follow-up of 67 patients who underwent OLIF. In addition, severe cage subsidence was more common in the unilateral fixation compared with bilateral fixation in lateral lumbar interbody fusion^45^. In this study, all patients were performed with bilateral percutaneous pedicle screw fixation after lumbar interbody fusion, and the surgeries were performed by a senior surgeon with over 10 years of experience who had performed more than 1000 cases of lumbar fusion surgery. Familiar with the anatomy of disc and good endplate preparation were beneficial with intraoperative protection of the bony endplate. In addition, the average BMI of all patients were at normal levels, and also without severe osteoporosis. All of these are the advantage factors that can help to prevent cage subsidence.

We found no significant difference in the total incidence of complications among the three groups; nonetheless, cerebrospinal fluid leakage occurred postoperatively in 4 cases in the Endo-TLIF group, which was higher than that in the other two groups. Based on previous literature, the incidence of dural tears with lumbar surgery ranges from 1 to 10.5%^46–49^. Significant risk factors contributing to cerebrospinal fluid leak include a high level of surgeon training, old patient age, degenerative spondylolisthesis, and previous history of surgery^47,50,51^. In this work, we noted that insertion of the nerve retractor was a risk factor promoting dural tears in the Endo-TLIF group. Several approaches to address cerebrospinal fluid leak have been documented. First, understanding its risk factors is fundamental for preventing dural tears. A minor dural tear discovered during surgery that is properly sutured and for which bed rest is ordered postoperatively is not a major issue. Fascial, muscular, and artificial grafts, fibrin glue, and subarachnoid drainage are all options in addition to direct suture closure^52^.

In the present study, OLIF led to a better effect in maintaining the lumbar lordosis angle and disc height during the follow-up. This was primarily attributed to the cage applied in OLIF, in which a larger cage that has a 5° lordosis angle is used, which gives stronger and more even support to the spine. Hung et al.^53^ compared the clinical outcomes between OLIF and MIS-TLIF in one single-level lumbar spondylosis. Their findings demonstrated that that OLIF can give superior outcome in restoring segmental lordosis and coronal imbalance. In another 2019 study, the researchers also found that OLIF can give superior outcome in restoring agittal disc angle in 1 year follow-up^54^. In addition, the cage geometry likely plays a role in alignment of lumbar spine after lumbar interbody fusion. For instance, in a study published by Godde et al.^55^, a total of 42 patients that underwent posterior lumbar interbody fusion. Twenty patients inserted with bullet-shaped cage had a mean decrease in segmental lordosis from 10° to 2° at L3-L4 and from 10° to 5°at L4-L5 while 22 patients inserted with wedge shaped cage had an average increase in segment lordosis from 4° to 7° at L3-L4 and 2° to 8° at L4-L5. Many studies on surgical outcomes of lumbar interbody fusion have suggested that having a cage covered in the anterior part of vertebral body could help with restoration of lumbar lordosis since the anterior portion of endplate is the strongest part^54,56,57^. In OLIF, the cage was positioned transversely over bilateral ends of the vertebral body, as it has done in the extreme lateral interbody fusion for patients with adult spinal deformity^58^, which restored quite well the coronal and sagittal alignments. This theory aligns with our result, as OLIF, with its wide cage being inserted in the relatively anterior part of vertebral body, showed better improvement in restoring segmental lordosis post-operatively. In line with previous research results, OLIF led to a better effect in maintaining the lumbar lordosis angle and disc height during the follow-up in our study.

Our research showed that all three minimally invasive lumbar fusion surgeries achieved a high fusion rate. However, the imaging evaluation results showed that the OLIF group had a significantly higher fusion rate. The placement direction of the cage and the amount of bone graft can both affect fusion status^59,60^. OLIF employs a larger cage, which helps the cage hold more bone-grafting granules. On the other hand, the cage is obliquely inserted into the intervertebral space in MIS-TLIF or Endo-TLIF, which causes weak support on the nonimplanted side and uneven stress in the intervertebral space. When the cage is horizontally inserted in OLIF, the distance between the cage and edge of the intervertebral disc is relatively similar, which contributes to the symmetrical distribution of stress in the intervertebral space and is conducive to promoting bone fusion. Wang et al.^60^ compared the stabilities imparted by the cages placed using an oblique and conventional posterior approaches in vitro study, their findings suggested that the parallel transverse cage provids better stability than oblique cage. LU et al.^61^ compared the biomechanical differences among TLIF, XLIF, and OLIF by the finite element analysis, their findings indicated that the OLIF induced fewer stress peaks in the cortical endplate and cancellous bone than did the TLIF procedure, which was beneficial for subsidence resistance and disc height and segmental angle maintenance.

For the first time, we retrospectively analysed the MIS-TLIF, OLIF, and Endo-TLIF approaches in the treatment of L4/5 degenerative spondylolisthesis. Based on research findings, OLIF achieved better short medium term clinical effects than MIS-TLIF and Endo-TLIF. OLIF as a minimally invasive technique with a wider range of clinical indications, and its advantages are obvious, but further evaluation is required to compare its operation-related data with that of traditional open surgery. Mobbs et al.^62^ insisted that OLIF is contraindicated in patients with severe central canal stenosis and high grade spondylolisthesis. Meanwhile, its intraoperative and postoperative complications including abdominal vascular injury, endplate damage and vertebral fracture, urethral injury, sympathetic chain injury should be pay great importance^44,63,64^. In addition, perfect technique and acquainted anatomical knowledge play important role in successful operation.

This work has compelling limitations. First of all, the study was retrospective in nature. There may be selection bias, as surgeons determined which surgery approach should be performed. In addition, the experience and mastery degree of skillsets of surgeon could have some impact on the results. Secondly, we used a relatively small sample size and short-term follow up. Additional prospective randomized controlled clinical trials and long-term follow-up results are necessary in the future. Lastly, it is regrettable that the cost considerations have not been inspected in our study. Additional detailed cost-effectiveness analysis is necessary for us in the future.

## Conclusion

In conclusion, although MIS-TLIF, OLIF, and Endo-TLIF techniques effectively treat patients with L4/5 degenerative spondylolisthesis, OLIF has more benefits, including less operative blood loss, shorter hospital stay, less drainage volume, efficacy for back pain, effective maintenance of lumbar lordosis angle and disc height, and higher fusion rates. OLIF should be the preferred surgical treatment for patients with L4/5 degenerative spondylolisthesis.

## Data Availability

The datasets used and/or analysed in the current study are available from the corresponding author on reasonable request.
